# Mucosa‐associated lymphoid tissue lymphoma with isolated endobronchial involvement

**DOI:** 10.1002/rcr2.672

**Published:** 2020-10-16

**Authors:** Ting‐Yu Liao, Chien‐Chin Lin, Chang‐Tsu Yuan, Ching‐Kai Lin, Chao‐Chi Ho

**Affiliations:** ^1^ Division of Pulmonary Medicine, Department of Internal Medicine National Taiwan University Hospital Taipei Taiwan; ^2^ Division of Hematology, Department of Internal Medicine National Taiwan University Hospital Taipei Taiwan; ^3^ Department of Laboratory Medicine National Taiwan University Hospital Taipei Taiwan; ^4^ Graduate Institute of Clinical Medicine National Taiwan University Taipei Taiwan; ^5^ Department of Pathology National Taiwan University Hospital Taipei Taiwan; ^6^ Department of Pathology National Taiwan University Cancer Center Taipei Taiwan; ^7^ Department of Medicine National Taiwan University Cancer Center Taipei Taiwan

**Keywords:** Bronchoscopy, MALT lymphoma, primary pulmonary lymphoma

## Abstract

Primary pulmonary lymphoma is an uncommon disease, and extranodal marginal zone lymphoma of mucosa‐associated lymphoid tissue (MALT) is the most common type of pulmonary lymphoma. The most frequent pattern observed in chest computed tomography (CT) is consolidation, followed by nodules and mass. The differentiation of pulmonary MALT lymphoma from other lung diseases is critical for disease management and treatment. However, pulmonary MALT lymphoma with isolated endobronchial manifestation has seldomly been reported. Here, we report a case of an elderly woman who presented with a four‐month history of cough, dyspnoea, and haemoptysis. Chest CT scan revealed left main bronchus narrowing without lung parenchymal lesion. Bronchoscopic examination was performed, and the diagnosis of primary pulmonary MALT lymphoma with isolated endobronchial involvement was made. She has been successfully treated with rituximab.

## Graphical abstract

We describe a case of primary pulmonary mucosa‐associated lymphoid tissue (MALT) lymphoma presented as an endobronchial tumor without lung parenchymal involvement.

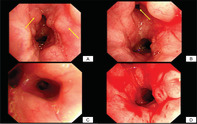

## Introduction

Primary pulmonary lymphoma (PPL) is defined by clonal lymphoid proliferation affecting lungs, including parenchyma and/or bronchi, without detectable extrapulmonary involvement at diagnosis or during the subsequent follow‐up period of three months [1]. The incidence of PPL is very low, representing only 0.5–1% of all lung neoplasm cases [2, 3].

Extranodal marginal zone lymphoma of mucosa‐associated lymphoid tissue (MALT) represents over 80% of PPL cases [2, 4]. Patients with pulmonary MALT lymphomas typically present with lung alveolar opacity and air bronchogram under radiological study [2]. Without lung parenchymal involvement, primary pulmonary MALT lymphoma with unique endobronchial involvement is rarely observed. We describe a case of primary pulmonary MALT lymphoma presented as an endobronchial tumour without lung parenchymal involvement.

## Case Report

**Figure 1:**
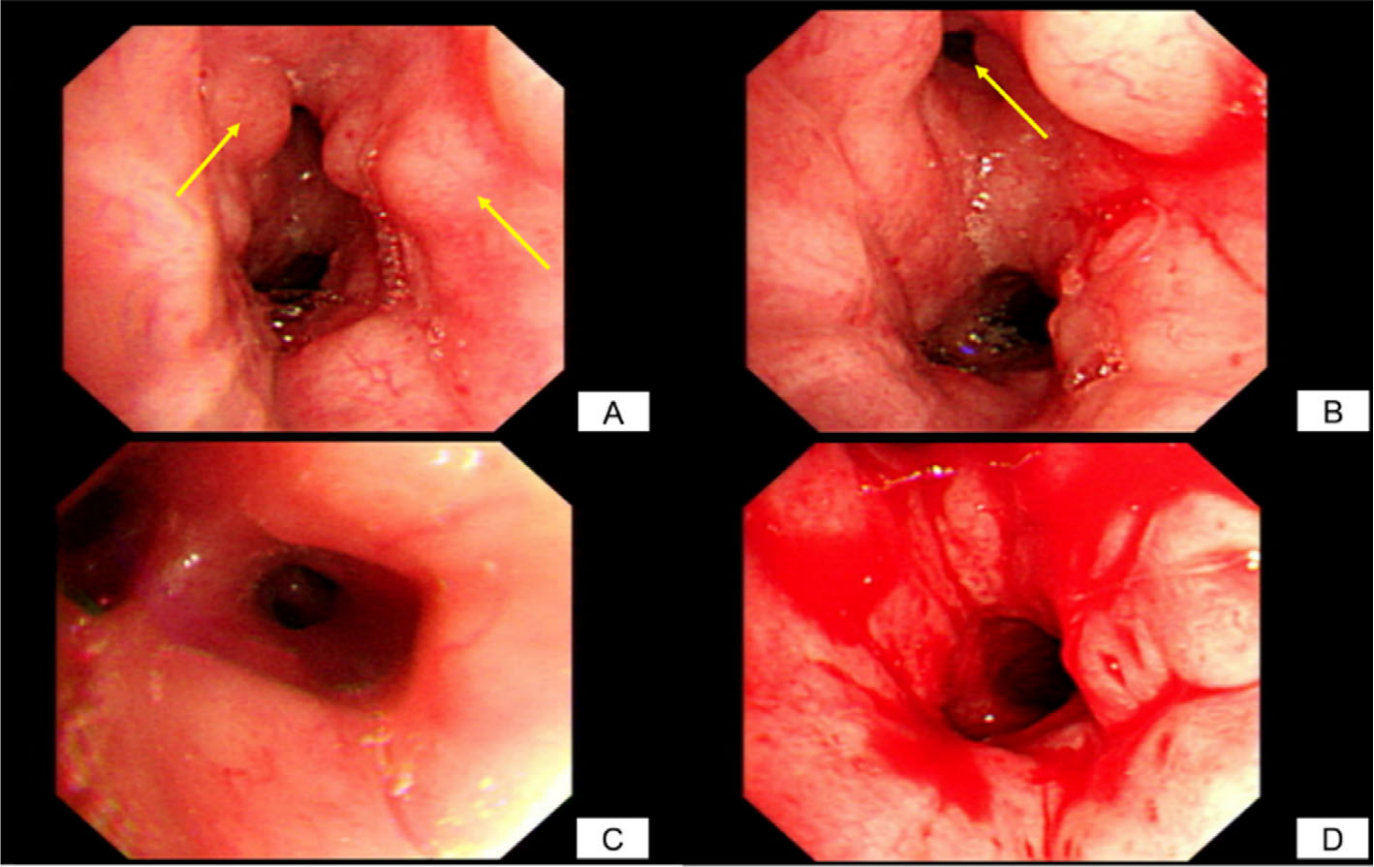
Bronchoscopic finding revealed multiple endobronchial nodules noted at the left main bronchus (A), causing left upper deviation orifice (B) narrowing. No endobronchial lesion was noted at the left upper deviation (C). It was an easy touch bleeding (D).

**Figure 2:**
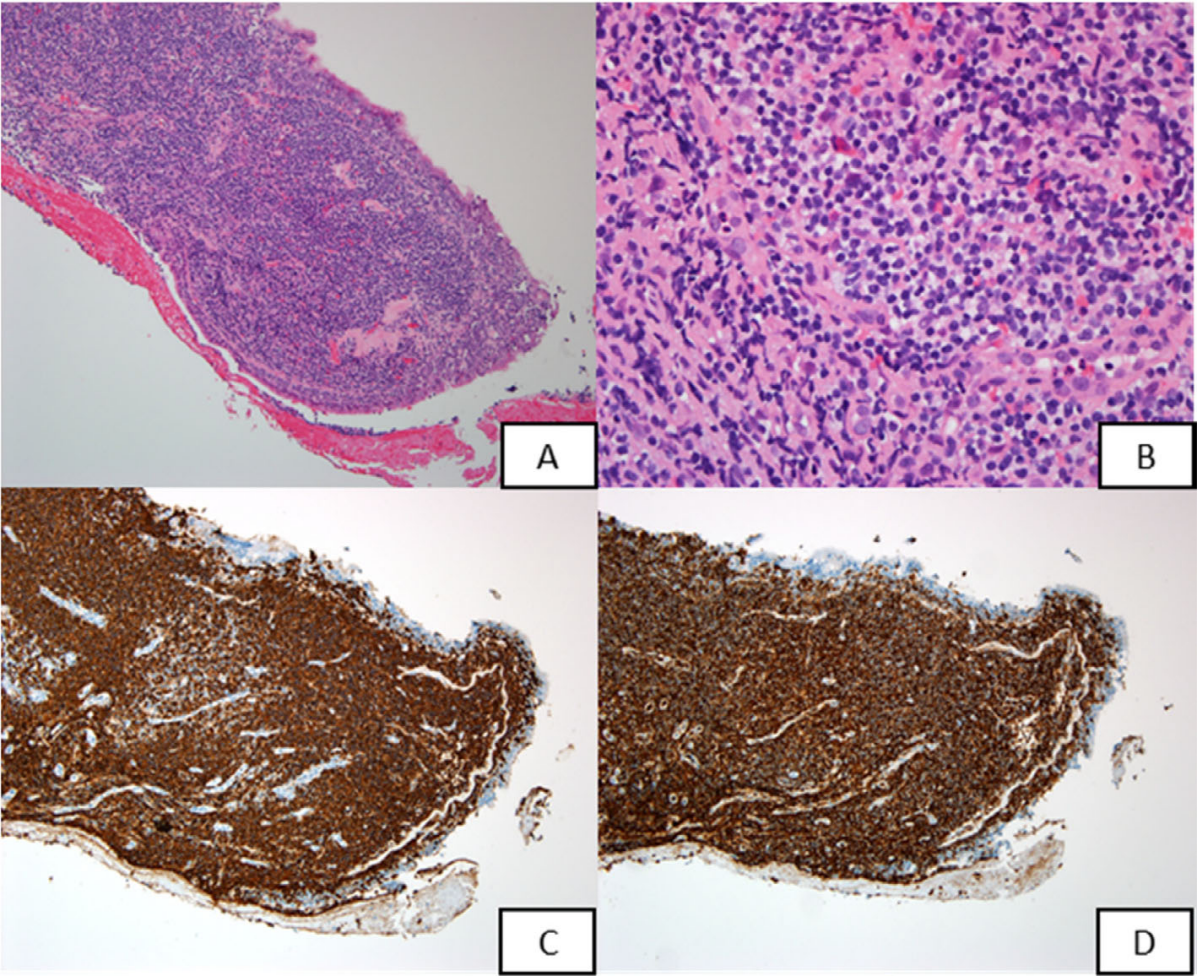
Pathology of endobronchial biopsy. There was atypical dense lymphoid infiltrates under respiratory epithelium (A, haematoxylin and eosin (H&E) stain, original magnification 100×). The lymphoid infiltrates were composed of small‐ to medium‐sized monocytoid cells (B, H&E stain, original magnification 400×). These lymphoid cells were positive for CD20 (C, CD20 immunostain, clone L26, Ventana (AZ, USA), original magnification 100×) and aberrantly express CD43 (D, CD43 immunostain, clone L60, Ventana (AZ, USA), original magnification 100×).

A woman of age 85 years presented at our clinic with a four‐month history of cough and dyspnoea accompanied by haemoptysis. There was no associated fever, weight loss, or chest pain. She had a medical history of hypertension and had been taking lacidipine. The respiratory rate was 18/min, heart rate was 71/min, body temperature was 36°C, and blood pressure was 187/88 mmHg. Chest auscultation disclosed left‐sided minimal wheezing. Other examinations including cardiovascular, abdominal, nervous, and musculoskeletal system did not reveal any abnormalities. The laboratory results showed normal haemogram, biochemistry, lactate dehydrogenase (LDH), and carcinoembryonic antigen (CEA) levels. Chest radiograph showed no obvious lung lesion initially. Chest computed tomography (CT) scan disclosed severe narrowing in the left main bronchus without lung parenchymal lesion. Bronchoscopy revealed mucosa nodularity with easy touch bleeding from the left main bronchus to the left upper deviation (Fig. 1). Biopsy revealed a low‐grade B‐cell lymphoma. The lymphoma cells were positive for CD20 and CD43, whereas they were negative for CD3, CD5, CD10, and cyclin D1 (Fig. 2). There was no bone marrow involvement. Whole body CT scan showed no obvious lymphadenopathy. Bronchial mucosa was the only involvement site. The diagnosis of pulmonary MALT lymphoma, Ann Arbor stage II, was made. Rituximab with 375 mg/m^2^ body surface area (BSA) was administered every four weeks without significant adverse effect. After the treatment, the patient was free of respiratory symptoms and is currently in good health.

## Discussion

MALT lymphoma is a lymphoma found in mucosal area, originating from post‐germinal centre memory B‐cells [1, 2]. It was first described in the gastrointestinal tract, and the most frequently affected organ is the stomach [1, 4]. *Helicobacter pylori* eradication induces remission in most patients with gastric MALT lymphoma [1, 4, 5]. Pulmonary MALT lymphomas represent over 80% of all PPL cases [2, 4]. Unlike gastric MALT lymphoma, no antigens triggering pulmonary MALT lymphoma have been identified to date [6].

The age of onset is usually 50–60 years with equal frequency in males and females, and MALT lymphoma very occasionally affects those younger than 30 years. Half of the patients are asymptomatic, and the disease is discovered as an incidental finding. Some patients present with cough, mild dyspnoea, chest pain, and haemoptysis [2, 4]. The first investigation is usually due to an abnormal chest image. Chest radiograph typically shows a chronic localized alveolar opacity, less than 5 cm in diameter, and an air bronchogram in nearly 50% of cases [2]. The CT scan is more sensitive than standard radiography [2]. MALT lymphomas exhibit various patterns of lung abnormality on CT scan, including consolidation with air bronchograms, nodules, mass, ground‐glass opacity, and diffuse interstitial lung disease patterns. The most common patterns are consolidation, followed by nodules and mass [2, 7]. The majority of MALT lesions are bilateral (60–70% of cases) and multiple (70–77% of cases). Hilar or mediastinal lymphadenopathy is present in approximately 30% of cases [2, 3]. Yoon et al. reviewed seven cases of endobronchial MALT lymphoma and classified the abnormalities detected by CT scan into three groups: solitary intraluminal nodule (57.1%), several tiny nodular protrusion (28.6%), and diffuse wall thickening (14.3%) [8].

Diagnosis of pulmonary MALT lymphoma is a challenge in the clinical practice because symptoms and radiological findings are always non‐specific. When the diagnosis of MALT lymphoma is considered, several assessments should be performed to exclude other possible conditions, including infection, inflammation, and malignancies. The diagnosis of MALT lymphoma is based on the identification of morphological, immunophenotypic, genotypic, and molecular features, including flow cytometry analysis of the tumour [9]. Tissue biopsy is the gold standard for diagnosis [2]. To use minimally invasive techniques, bronchoscopy or CT‐guided needle biopsy is preferred, depending on the lesion's site. Borie et al. reviewed 63 cases with pulmonary MALT lymphoma. Pathological diagnosis of pulmonary MALT lymphoma was obtained by minimally invasive procedures, including bronchoscopic biopsies and CT‐guided percutaneous transthoracic biopsies in 45 cases (71.4%), and 38 cases (60.3%) were diagnosed by bronchial or transbronchial biopsies [10]. Kawaguchi et al. reviewed 20 cases with endobronchial MALT lymphoma [9]. Among these, 61% exhibited several nodular protrusions, 22% solitary intraluminal nodules, and 17% diffuse wall thickening [9]. The diagnostic yield of bronchial, and especially transbronchial, biopsy is higher when it targets visible endobronchial lesions or radiographic abnormalities [11]. In our case, bronchoscopic biopsy seems to be the only way to obtain reliable diagnostic data. Besides, transbronchial cryobiopsy is a new method that allows to obtain larger samples without crushed artefacts; the samples obtained with this approach can be 20 times bigger than those obtained by conventional transbronchial lung biopsy [12]. Incidences of complications including bleeding and pneumothorax from transbronchial cryobiopsy appear slightly higher compared with those from conventional transbronchial biopsy, but without any increase in mortality risk [13]. To our knowledge, there are no large studies evaluating the utility of transbronchial cryobiopsy in pulmonary lymphoma.

There is no consensus for the choice of treatment, and various regimens including radiotherapy, surgery, and chemotherapy have been proposed. If the patient is asymptomatic, close observation is suggested [5]. For patients with disseminated MALT lymphoma, rituximab treatment in combination with chemotherapy appears as the most appropriate choice [4]. On the whole, the prognosis for patients with MALT lymphoma is excellent. According to a recent report, the 10‐year recurrence‐free, overall survival, and cause‐specific survival rates were 76%, 87%, and 98%, respectively [4]. In our case, the patient received seven cycles of rituximab. Serial images showed complete metabolic response. She has been examined regularly at follow‐up visits.

In conclusion, the patient presented with a tumour with limited localization to the bronchial trees without lung parenchyma involvement, and bronchoscopy for tissue analysis and accurate diagnosis was of critical importance. Because the prognosis for patients with pulmonary MALT lymphoma is good, patients should be examined closely and treated accordingly.

### Disclosure Statement

Appropriate written informed consent was obtained for publication of this case report and accompanying images.

## References

[1] Cadranel J , Wislez M , and Antoine M . 2002 Primary pulmonary lymphoma. Eur. Respir. J. 20:750–762. 12358356 10.1183/09031936.02.00404102

[2] Borie R , Wislez M , Antoine M , et al. 2016 Pulmonary mucosa‐associated lymphoid tissue lymphoma revisited. Eur. Respir. J. 47:1244–1260. 26797028 10.1183/13993003.01701-2015

[3] Hare SS , Souza CA , Bain G , et al. 2012 The radiological spectrum of pulmonary lymphoproliferative disease. Br. J. Radiol. 85:848–864. 22745203 10.1259/bjr/16420165 PMC3474050

[4] Zucca E , and Bertoni F . 2016 The spectrum of MALT lymphoma at different sites: biological and therapeutic relevance. Blood 127:2082–2092. 26989205 10.1182/blood-2015-12-624304

[5] Kim JS , Chung SJ , Choi YS , et al. 2007 *Helicobacter pylori* eradication for low‐grade gastric mucosa‐associated lymphoid tissue lymphoma is more successful in inducing remission in distal compared to proximal disease. Br. J. Cancer 96:1324–1328. 17406363 10.1038/sj.bjc.6603708 PMC2360178

[6] Isaacson PG . 1999 Mucosa‐associated lymphoid tissue lymphoma. Semin. Hematol. 36:139–147. 10319382

[7] Deng W , Wan Y , and Yu JQ . 2019 Pulmonary MALT lymphoma has variable features on CT. Sci. Rep. 9:8657. 31209274 10.1038/s41598-019-45144-9 PMC6572828

[8] Yoon RG , Kim MY , Song JW , et al. 2013 Primary endobronchial marginal zone B‐cell lymphoma of bronchus‐associated lymphoid tissue: CT findings in 7 patients. Korean J. Radiol. 14:366–374. 23483549 10.3348/kjr.2013.14.2.366 PMC3590354

[9] Kawaguchi T , Himeji D , Kawano N , et al. 2018 Endobronchial mucosa‐associated lymphoid tissue lymphoma: a report of two cases and a review of the literature. Intern. Med. 57:2233–2236. 29526933 10.2169/internalmedicine.0150-17 PMC6120826

[10] Borie R , Wislez M , Thabut G , et al. 2009 Clinical characteristics and prognostic factors of pulmonary MALT lymphoma. Eur. Respir. J. 34:1408–1416. 19541720 10.1183/09031936.00039309

[11] Cordier JF , Chailleux E , Lauque D , et al. 1993 Primary pulmonary lymphomas. A clinical study of 70 cases in nonimmunocompromised patients. Chest 103:201–208. 8417879 10.1378/chest.103.1.201

[12] Poletti V , Gurioli C , Piciucchi S , et al. 2015 Intravascular large B cell lymphoma presenting in the lung: the diagnostic value of transbronchial cryobiopsy. Sarcoidosis Vasc. Diffuse Lung Dis. 31:354–358. 25591148

[13] Yap E , and Low I . 2017 Bronchoscopic transbronchial cryobiopsy diagnosis of recurrent diffuse large B‐cell lymphoma in the lung: a promising new tool? J. Bronchology Interv. Pulmonol. 24:e22–e23. 28323739 10.1097/LBR.0000000000000354 PMC5367508

